# Mid-Term Outcomes of a Next-Generation Modular Acetabular System in Primary and Revision Total Hip Arthroplasty

**DOI:** 10.3390/jcm15114258

**Published:** 2026-05-31

**Authors:** Garrett Ruff, Laith Bahlouli, Anzar Sarfraz, Farouk Khury, Diren Arsoy, Claudette Lajam, Vinay K. Aggarwal

**Affiliations:** 1 Department of Orthopedic Surgery, NYU Langone Health, New York, NY 10016, USA; 2 The Ruth and Bruce Rappaport Faculty of Medicine, Technion—Israel Institute of Technology, Haifa 3525433, Israel

**Keywords:** total hip arthroplasty, revision hip arthroplasty, acetabular component, modular cup, dual-mobility, implant survivorship, HOOS, JR

## Abstract

**Background/Objectives**: Total hip arthroplasty (THA) is a common orthopedic procedure, and with projected growth in both primary and revision surgical volumes, robust implant performance data is necessary to inform surgical decision-making. To ensure successful outcomes in primary THA (pTHA) and revision THA (rTHA), surgeons need versatile implant systems that can address patient-specific surgical challenges. This study aimed to evaluate the outcomes of a next-generation acetabular system used for various indications in both pTHA and rTHA. **Methods**: We retrospectively reviewed 319 patients who underwent either pTHA or rTHA using a modern acetabular system at a single urban academic center between 2014 and 2023 with at least 18 months of follow-up. Baseline characteristics and the patient-reported Hip Disability and Osteoarthritis Outcome Score, Joint Replacement (HOOS, JR) were collected. A total of 284 patients who underwent pTHA and 35 patients who underwent rTHA were included. Median follow-up was 2.6 years (range: 1.5–8.4 years). **Results**: The most common indication was osteoarthritis (90%) for pTHA and instability (46%) for rTHA. Most rTHAs utilized a dual-mobility construct (74%), compared to pTHAs (22%). There were ten all-cause acetabular revisions in the entire cohort (eight in pTHA, two in rTHA), four of which were aseptic (three in pTHA, one in rTHA). All-cause and aseptic acetabular survivorship of the pTHA cohort was 97.2% and 98.7%, respectively, and of the rTHA cohort was 94.3% and 97.1%, respectively. Improvement in the median HOOS, JR score was 21.5 points at one year and 25.5 points at two years among pTHAs. **Conclusions**: The findings with this system support adequate mid-term acetabular component survivorship in pTHA and rTHA, along with clinically meaningful functional improvement following pTHA. Given the retrospective, observational nature of this study, further prospective research with extended follow-up and larger sample sizes, particularly in the rTHA cohort, is needed to better assess long-term outcomes.

## 1. Introduction

Total hip arthroplasty (THA) is a common and highly successful orthopedic procedure, and the demand for THA is anticipated to significantly rise due to an aging population, with projections estimating nearly triple the surgical volume by 2040 compared to 2019, amounting to approximately 1.5 million annual procedures [1,2,3]. The distribution of THA procedures varies substantially by indication and patient population, with primary procedures comprising the large majority of volume; however, revision THA (rTHA) accounts for a distinct and growing proportion, and exhibits important differences in surgical complexity, patient demographics, and clinical outcomes compared to primary procedures [1,2,3,4,5]. Despite generally favorable patient outcomes, the incidence of rTHA is projected to increase by 2–3% annually, driven primarily by the increasing number of primary THA (pTHA) procedures [4,5]. As rTHA is associated with increased healthcare costs, higher complication incidence, and diminished implant survival, orthopedic surgeons must utilize versatile implant systems to optimize patient outcomes and minimize the risk of revision and subsequent re-revision [6,7,8,9,10,11].

Approximately 80% of rTHAs are performed due to aseptic mechanical failure, with instability and aseptic loosening representing the predominant indications, each accounting for roughly 20% of all revision procedures [12,13]. Mechanical failure, particularly aseptic loosening, arises from inadequate fixation of implants during primary THA, periprosthetic osteolysis, and insufficient osseointegration at the bone–implant interface [14,15]. Highly porous acetabular cups have demonstrated reduced incidence of aseptic loosening by improving osseointegration at the bone–implant interface [16,17]. In primary procedures with compromised bone quality and revision cases with bony defects, the use of acetabular screws can further decrease loosening incidence [18,19]. Additionally, strategies such as large femoral heads, dual-mobility articulations, and constrained polyethylene liners effectively reduce instability [20]. Therefore, orthopedic surgeons require implant systems offering highly porous surface materials, variability in screw-hole number, compatibility with monoblock and dual-mobility articulations, and interchangeability among different polyethylene liners and femoral head sizes.

A next-generation acetabular system for primary and revision THA was introduced, with versatile features and compatible sub-devices, including options for both fixed-bearing monoblock, dual-mobility, and constrained liners. However, little evidence is available regarding the clinical and functional outcomes of this acetabular system in primary and revision THA populations. This study aims to investigate mid-term postoperative outcomes of this next-generation acetabular system at our institution and compare these outcomes to previously published data from other hip arthroplasty systems.

## 2. Materials and Methods

### 2.1. Cohort Selection and Eligibility Criteria

**Figure 1:**
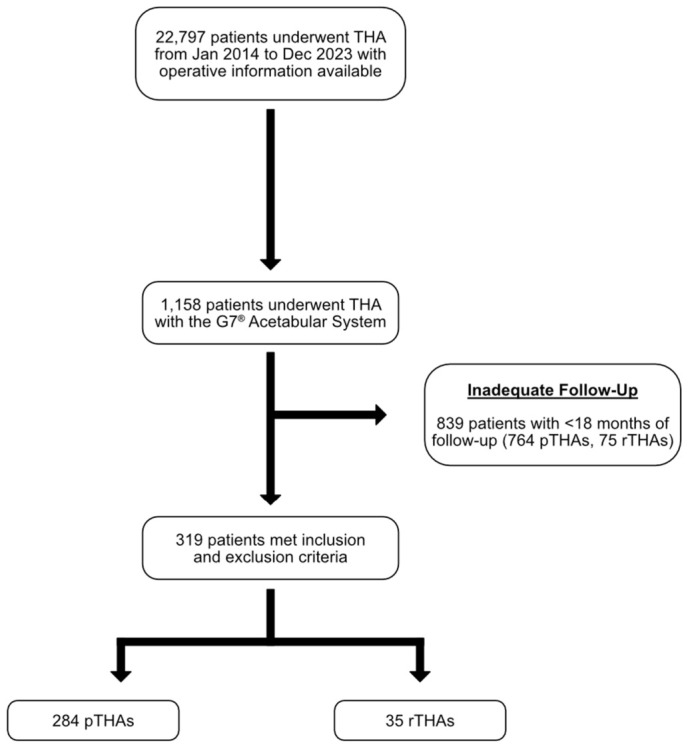
Flow chart depicting patient flow with inclusion and exclusion criteria.

A retrospective review was conducted of all THA procedures at a single, urban, academic, tertiary care center between 1 January 2014, and 31 December 2023. Patients undergoing either pTHA or rTHA with a next-generation acetabular system (G7^®^ Acetabular System [Zimmer Biomet, Warsaw, IN, USA]) and an associated liner from the same company were included for analysis. Patients with less than 18 months of clinical follow-up, defined as a clinic visit with our orthopedic department, were excluded to best represent mid-term outcomes [21,22,23]. Patients lacking operative notes or information regarding implants utilized in their procedure were also excluded. Patient flow is depicted in Figure 1. Institutional review board (IRB) approval was obtained before initiating this study: i17-01223.

### 2.2. Study Design and Patient Characteristics

Patient demographics and clinical characteristics, including age, sex, body mass index (BMI), and American Society of Anesthesiologists (ASA) score, were collected from electronic medical records. Perioperative variables were also collected, including implants used and surgical indication. Patients were categorized into primary versus revision THA cohorts. The use of dual-mobility constructs was determined from operative records. The utilization of dual-mobility constructs is a surgeon-specific decision, reflecting both individual surgeon preference and the specific indications and anatomy of each patient.

The primary outcome assessed was the occurrence of acetabular revision or re-revision surgery for either septic or aseptic indications. Survivorship free from all-cause and aseptic acetabular revision and re-revision for pTHAs and rTHAs, respectively, was analyzed. Additionally, functional outcomes in the pTHA cohort were evaluated using the Hip Disability and Osteoarthritis Outcome Score for Joint Replacement (HOOS, JR), routinely collected at our institution at the preoperative visit, and at each postoperative visit, which at our institution routinely occur at the two-week, six-week, six-month, one-year, and two-year periods. The HOOS, JR score is collected via an electronic survey that the patient completes before their visit. The response rate in the rTHA cohort was very low and thus not included in our analysis.

### 2.3. Data Analyses

Categorical variables were presented as count (percentage) and continuous variables as median [range] for follow-up duration or median [interquartile range]. All continuous variables were non-normally distributed by the Shapiro–Wilk test (*p* < 0.05). Kaplan–Meier analysis was performed to determine acetabular survivorship free from all-cause and aseptic revision for pTHA and rTHA separately. Significance was set at *p* < 0.05. All statistical analyses were performed with R version 4.2.0 (R Foundation for Statistical Computing; Vienna, Austria).

## 3. Results

### 3.1. Baseline Characteristics

A total of 319 patients who underwent THA with implantation of a next-generation acetabular cup were included, representing 284 primary THAs (89.0%) and 35 revision THAs (11.0%). Median follow-up in the cohort was 2.6 years, with slightly longer follow-up in the revision cohort than in the primary (3.2 vs. 2.6 years). There were no other clinically significant differences in baseline characteristics between primary and revision groups. A majority of revision procedures utilized a dual-mobility construct unlike in primaries (74.3% vs. 22.2%) (Table 1).

The majority of primary THAs were indicated for primary osteoarthritis (89.8%), with the remainder of procedures indicated for dysplasia (4.9%), avascular necrosis (1.8%), inflammatory arthritis (1.8%), post-traumatic arthritis (1.4%), and septic arthritis (0.4%). Conversely, revision THAs had a variety of surgical indications, most commonly instability (45.7%), periprosthetic joint infection (22.9%), and aseptic loosening (14.3%) (Table 2 and Table 3).

### 3.2. Revision and Re-Revision

**Figure 2:**
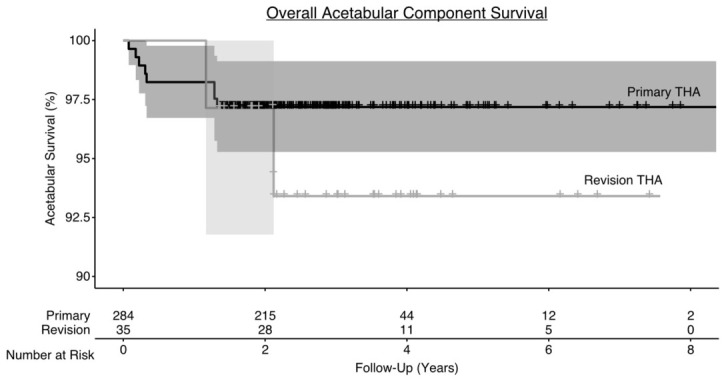
Kaplan–Meier all-cause acetabular survivorship stratified by index procedure. The shaded region represents the 95% confidence interval.

**Figure 3:**
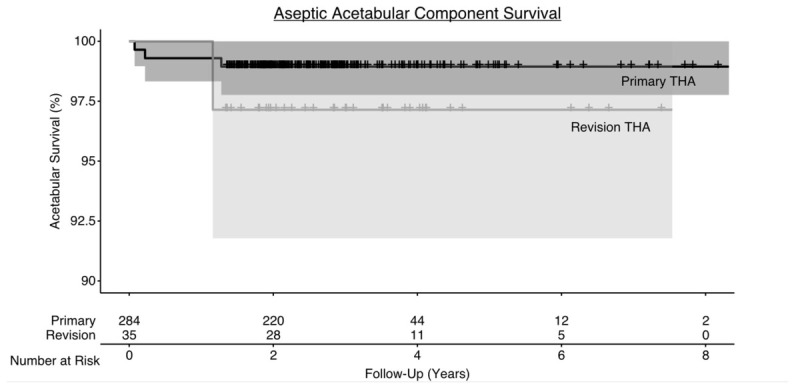
Kaplan–Meier aseptic acetabular survivorship stratified by index procedure. The shaded region represents the 95% confidence interval.

Of 284 primary THAs, eight patients required acetabular revision, five of which were due to periprosthetic joint infection (PJI). Two of the remaining acetabular revisions were due to recurrent dislocations requiring a revision acetabular cup and constrained liner implant. The final revision was after a complex primary THA in a patient with developmental dysplasia of the hip, acetabular deformity, and subchondral collapse of the femoral head. This patient suffered from failure of acetabular screw fixation and aseptic loosening of the acetabular component one month postoperatively, requiring acetabular cup revision. All-cause and aseptic acetabular survivorship of the pTHA cohort at latest follow-up was 97.2% and 98.7%, respectively (Figure 2 and Figure 3).

Conversely, in the revision THA cohort, two acetabular re-revisions occurred, one due to PJI and one due to aseptic loosening of the acetabular component with subchondral insufficiency fractures after a postoperative course complicated by recurrent falls.

### 3.3. Functional Outcomes

For patients who underwent primary THA, HOOS, JR scores improved substantially, increasing from a preoperative median of 51.5 points to a median of 73.0 points at one year and 77.0 at two years postoperatively—exceeding the established MCID threshold of approximately 20 points, indicating clinically meaningful improvement [24]. With a response rate well below 50%, the revision THA cohort functional outcomes were not analyzed (Table 4).

## 4. Discussion

This study demonstrates adequate mid-term outcomes for a next-generation acetabular cup system in both primary and revision THA. We observed a high all-cause acetabular component survivorship of 97.2% and an aseptic survivorship of 98.7% at latest follow-up among primary THAs. Notably, of the eight acetabular revisions in the pTHA cohort, only three had aseptic indications. Furthermore, pTHA patients exhibited a substantial improvement in hip function. This is evidenced by an increase of 21.5 points from the median HOOS, JR preoperative score to the median one-year score, and a marked 25.5-point increase to the median two-year score.

A diverse array of acetabular cup systems currently exists, each with documented performance metrics regarding survivorship and complications. Uncemented press-fit cups, for instance, have widely demonstrated favorable long-term outcomes, with national arthroplasty registry data reporting survivorship of 95% at 10 years and 91% at 18 years [16,25,26,27]. Specific designs have exhibited survivorship free from aseptic acetabular revision as high as 94% at 22 years in certain patient populations [25]. Porous and three-dimensional manufactured designs have also demonstrated strong mid-term outcomes, with 98.4% aseptic survivorship at a mean follow-up of 7.3 years [28]. Our 98.7% aseptic acetabular survivorship among pTHAs favorably compares against the performance of many other well-documented implants [25,27,29,30]. Notably, out of the ten acetabular revisions seen in this cohort, only two cases were due to aseptic loosening: one in a patient with developmental hip dysplasia, who suffered from failed acetabular screw fixation and early cup loosening, and one with subchondral insufficiency fractures following recurrent falls. This low incidence of aseptic revision, in the context of complex primary THA and insufficiency fractures, further supports the robustness of this implant-bone interface and its successful osseointegration.

Dual-mobility acetabular cups, increasingly utilized for their reported ability to reduce dislocation incidence, have shown excellent long-term survivorship in previous retrospective studies [29,30]. Second-generation uncemented dual-mobility cups have achieved 99.2% survivorship free from acetabular revision at ten and fifteen years after primary THA, although some studies report less favorable survivorship of 95.6% and 85.0% at 14 and 17 years respectively [29,31]. Notably, some modular, dual-mobility implants have suffered from intraprosthetic dislocations, representing an additional mode of failure within these designs [32,33,34]. Importantly, no intraprosthetic dislocations were observed in this cohort, while only two cases of revision due to instability were noted in the pTHA cohort, each after implantation of a monoblock construct. The adaptability of the investigated next-generation implant was particularly advantageous in addressing complex primary and revision scenarios where dual-mobility was indicated, including developmental hip dysplasia, inflammatory osteoarthritis, leg length discrepancy, and most notably, instability and aseptic loosening.

Instability following primary THA is a leading cause of revision surgery and was the primary indication for rTHA (45.7%) in this cohort. The substantial utilization of dual-mobility constructs (74.3%) in these revision cases is consistent with widely incorporated clinical strategies aimed at mitigating dislocation risk in patients prone to instability [35,36,37,38,39]. This next-generation system’s capacity to accommodate dual-mobility articulations appears potentially beneficial in these demanding clinical contexts, though the small rTHA cohort (*n* = 35) limits definitive conclusions regarding implant versatility in complex revision settings. The low rate of aseptic re-revision in the rTHA cohort (*n* = 1) is encouraging; however, given the limited sample size and mid-term follow-up, this finding necessitates prospective, adequately powered studies to confirm the implant’s performance in rTHA.

Beyond objective implant survivorship, the functional outcomes reported by patients were clinically significant. The observed increase of 21.5 and 25.5 points in the median HOOS, JR score from preoperative baseline to one year and two years, respectively, signifies a substantial improvement in hip-related pain and function [40,41]. The Centers for Medicare and Medicaid Services define a threshold of “substantial clinical benefit” as a 22-point increase in the HOOS, JR score after THA [24]. These patient-reported outcomes underscore the efficacy of this acetabular system in restoring patients’ capacity for daily activities and enhancing their overall quality of life following primary THA. The observed improvements at one and two years were also comparable to those reported in the literature [42]. While comprehensive patient-reported outcome data for the revision cohort were limited, the robust improvements documented in the primary THA group provide compelling evidence of the functional benefits associated with the implementation of this acetabular system.

This study is not without limitations. Firstly, its retrospective, single-center design may inherently introduce selection bias and limit the generalizability of the findings to broader patient populations and diverse clinical settings. These findings are observational in nature, as this study lacks a control group or matched cohort; comparisons with other implant systems are indirect and cross-study in design. Accordingly, these results should be considered hypothesis-generating and interpreted in the context of the study’s retrospective, single-center limitations. Secondly, while the median follow-up of 2.6 years provides valuable mid-term data, a longer follow-up duration would be beneficial to fully assess the long-term survivorship and complication incidence of this next-generation acetabular system, as implant longevity is a critical factor in THA. Notably, while excluding patients with less than 18 months of follow-up best indicates mid-term outcomes, it introduces the potential for attrition bias in those patients who did not follow up, either due to positive outcomes limiting the patient’s desire for follow-up or negative outcomes seen elsewhere. Finally, the relatively small sample size of the revision THA cohort (*n* = 35) compared to the primary THA cohort (*n* = 284) may limit the ability to detect subtle differences or less common complications specific to revision procedures.

While the addition of HOOS, JR scores provides important information on patient satisfaction, response rates were below 50% at each time point, with the rTHA cohort well below 50%, precluding analysis of functional outcomes in this cohort. Therefore, there is a high risk for response bias in our results, and our study’s assessment of the rTHA cohort is limited to survivorship analysis. Future studies should aim to utilize prospective collection and electronic outcome platforms, which should be helped by the Centers for Medicaid and Medicare policy to require patient-reported outcome measurement collection in all total joint arthroplasties [43].

## 5. Conclusions

This retrospective study reports mid-term outcomes with an adaptable acetabular system in both primary and revision THA. In pTHA, the high all-cause and aseptic acetabular component survivorship, coupled with marked improvements in patient-reported functional scores, highlights the adequate mid-term performance of this next-generation implant. Its utility in challenging revision cases, particularly those involving instability and aseptic loosening, and low re-revision incidence further supports its applicability in complex settings, though these findings require confirmation in larger, prospective cohorts. Further prospective research with larger sample sizes and extended follow-up is recommended to fully elucidate the long-term efficacy and broader applicability of this system.

## Figures and Tables

**Table 1:** Patient demographics.

Parameter	Primary THA (*n* = 284)	Revision THA (*n* = 35)
Age (years), median [IQR]	65.0 [57.0, 72.0]	67.0 [60.0, 73.0]
Men, *n* (%)	101 (35.6)	15 (42.9)
BMI (kg/m^2^), median [IQR]	28.7 [25.1, 33.2]	28.2 [24.9, 32.1]
Dual-Mobility, *n* (%)	63 (22.2)	26 (74.3)
ASA Class, *n* (%)		
I	14 (4.9)	0 (0.0)
II	159 (56.0)	20 (57.1)
III	104 (36.6)	12 (34.3)
IV	7 (2.5)	3 (8.6)
Follow-Up Duration (years), median [range]	2.6 [1.5–8.4]	3.2 [1.5–7.6]

**Table 2:** Surgical indications for primary total hip arthroplasty.

Indication, *n* (%)	Primary THA (*n* = 284)
Avascular necrosis	5 (1.8)
Dysplasia	14 (4.9)
Septic arthritis	1 (0.4)
Osteoarthritis	255 (89.8)
Post-traumatic	4 (1.4)
Inflammatory	5 (1.8)

**Table 3:** Surgical indications for revision total hip arthroplasty.

Indication, *n* (%)	Revision THA (*n* = 284)
Aseptic loosening	5 (14.3)
Conversion from hemiarthroplasty	2 (5.7)
Instability	16 (45.7)
Polyethylene wear	2 (5.7)
Leg-length discrepancy	1 (2.9)
Periprosthetic joint infection	8 (22.9)
Periprosthetic fracture	1 (2.9)

**Table 4:** HOOS, JR for primary total hip arthroplasty.

Median [IQR], (*n*)	Primary THA (*n* = 284)
Preoperative	51.5 [42.7, 59.0] (91)
6-week	70.0 [61.3, 82.0] (115)
1-year	73.0 [60.4, 85.0] (120)
2-year	77.0 [62.2, 100.0] (92)

## Data Availability

The original contributions presented in this study are included in the article. Further inquiries can be directed to the corresponding author.

## Abbreviations

The following abbreviations are used in this manuscript:

THA	Total hip arthroplasty
rTHA	Revision total hip arthroplasty
pTHA	Primary total hip arthroplasty
IRB	Institutional review board
BMI	Body mass index
ASA	American Society of Anesthesiologists
HOOS, JR	Hip Disability and Osteoarthritis Outcome Score for Joint Replacement
kg	Kilogram
m	Meter
IQR	Interquartile range
PJI	Periprosthetic joint infection
